# Epidermoid Cyst in the Appendix: A Rare Entity

**DOI:** 10.7759/cureus.22321

**Published:** 2022-02-17

**Authors:** Yu Ya Nway, Raj Shah, Jignesh Parikh, Ernesto Robalino Gonzaga

**Affiliations:** 1 Internal Medicine, University of Central Florida HCA Healthcare Graduate Medical Education, Greater Orlando, USA; 2 Pathology, Orlando Veterans Affairs Medical Center, Orlando, USA; 3 Internal Medicine, University of Central Florida College of Medicine, Orlando, USA; 4 Internal Medicine, University of Central Florida, Orlando, USA

**Keywords:** benign, appendicitis, pathology, epidermoid cyst, appendix

## Abstract

Epidermoid cysts are benign cystic lesions that are usually found in the skin. They can also be found in the organs of the gastrointestinal system. Here, we report a rare case of epidermoid cyst of the appendix which has been published in only three cases in the literature. They can be either congenital or acquired. Congenital epidermoid cysts are related to the inclusion of ectodermal elements at the time of neural groove closure. Acquired cysts are thought to be due to trauma or iatrogenic implantation of the epidermis in locations favorable to growth during surgery. Diagnosis is mainly by histopathological examination of the tissue sample. Complete excision of the cyst with the walls intact is considered curative.

## Introduction

Epidermoid cysts are benign cystic lesions that are usually found in the skin. They are most commonly located on the face, neck, and trunk [1]. They have been reported in other organs such as the caecum, liver, spleen, kidney, pancreas, and gonads. Gastrointestinal epidermoid cysts are uncommon, especially in the appendix, with only three case reports published in the literature. Here, we report a rare case of an epidermoid cyst of the appendix. 

## Case presentation

**Figure 1:**
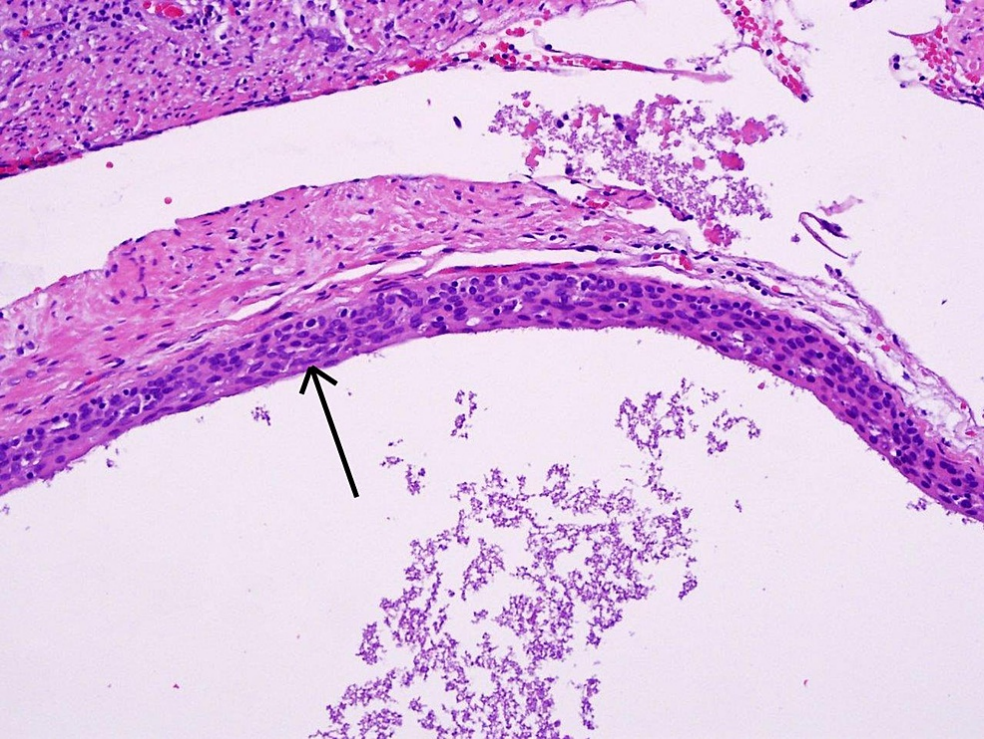
Hematoxylin & Eosin-stained sections from the appendix showed a squamous epithelial lined benign cystic lesion

**Figure 2:**
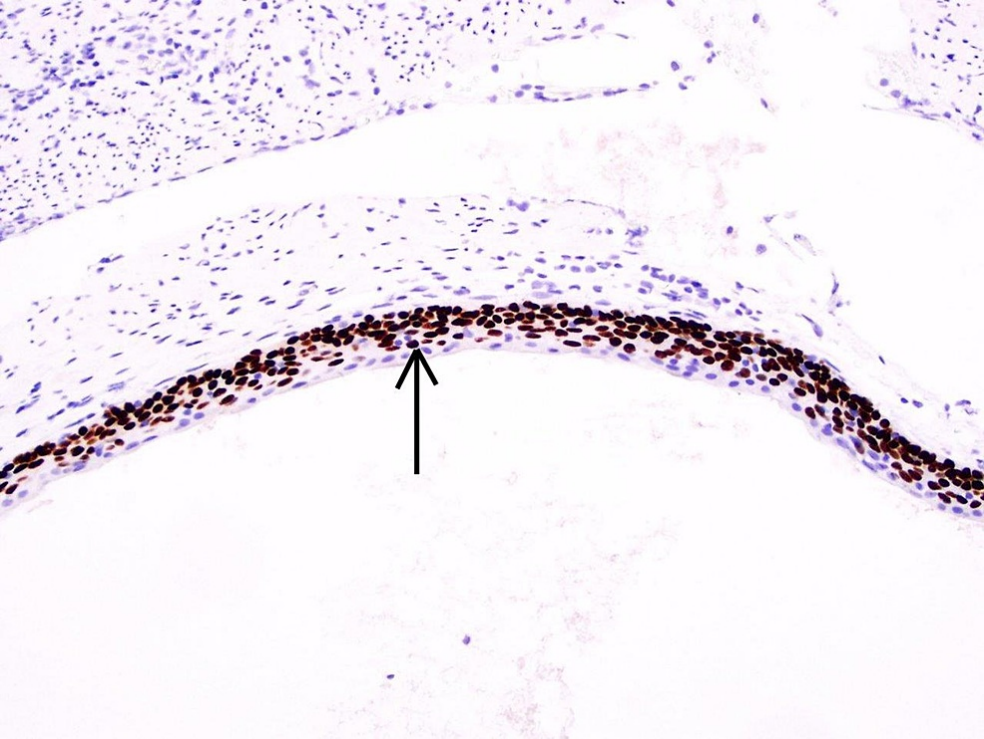
Cytokeratin immunostaining is positive within the squamous lining of the cyst

A 34-year-old gentleman with a past medical history of hypertension, hyperlipidemia, obstructive sleep apnea presented with a one-week history of abdominal pain. The pain started in the periumbilical region and radiated to right lower quadrant. It was associated with anorexia, nausea and diarrhea. There was no history of abdominal trauma or surgery. Physical examination was significant for right lower quadrant and suprapubic tenderness. There was no fever or leukocytosis. Computed Tomography (CT) scan of the abdomen and pelvis revealed a dilated appendix with peri-appendiceal fat stranding, thickening of adjacent peritoneal reflection and multiple enlarged mesenteric and pericolic lymph nodes. With the diagnosis of acute appendicitis, laparoscopic appendectomy was performed. On gross examination, the appendix was dilated at 10 mm in diameter. The serosa was tan-pink and focally smooth with scattered adhesions and tan-white exudate-like material at the junction of the serosa and the appendiceal fat. The mucosa was firm and tan-white, with an average wall thickness of 1 mm. No fecaliths or obvious transmural perforation sites were appreciated grossly. There was a benign cystic lesion that was removed and sent for biopsy. Histopathological examination revealed cyst wall lined by squamous epithelium (Figure 1) immunostaining for cytokeratin (Figure 2). The final pathological diagnosis confirmed epidermoid cyst of the appendix. Postoperatively, the patient had full recovery and was discharged home.

## Discussion

An epidermoid cyst is a benign encapsulated, subepidermal nodule filled with keratin material [1]. It is also known as a sebaceous cyst which is a misnomer as it does not consist of sebaceous material. Common synonyms include infundibular cyst, epidermal cyst, and epidermal inclusion cyst [1]. Epidermoid cysts are the most common cutaneous cyst, commonly found in the head and neck region [2]. In the gastrointestinal and digestive systems, epidermoid cysts have been reported in the caecum, liver, and pancreas [3-6]. An appendiceal epidermoid cyst is a rare entity that has been reported in only three cases. The histogenesis of epidermoid cysts in the gastrointestinal and digestive systems is uncertain. They can be congenital or acquired. Congenital epidermoid cysts are related to the inclusion of ectodermal elements at the time of neural groove closure [7]. Acquired cysts are thought to be due to trauma or iatrogenic implantation of the epidermis in locations favorable to growth during surgery [8]. Clinical symptoms of intestinal epidermoid cysts may vary from slow-growing intra-abdominal masses, vague abdominal discomfort to acute abdominal pain [9]. Diagnosis is made by histopathological examination as imaging findings are nonspecific. On review of the literature, all the reported cases of appendiceal epidermoid cysts including our case were thought to be congenital as there was no previous history of trauma or abdominal surgeries. One case presented with a prolonged history of asymptomatic non-tender right lower quadrant mass [10], while the other two presented as acute abdominal pain as in our case [11]. This is an incidental and histologic finding, yet a rare cause of acute abdomen. Complete excision of the cyst with the walls intact is considered curative although recurrence after incomplete excision is possible. Epidermoid cysts are usually benign lesions; however, histopathologic examination of the appendix is required to rule out malignancy as squamous cell carcinoma can arise from epidermoid cyst [12].

## Conclusions

Epidermoid cysts in the appendix are unique pathology with only three cases published in the literature. It can present as asymptomatic abdominal mass as well as acute abdominal pain. Although it is benign and rare entity, clinician should have high index of suspicion for this diagnosis since it can present as an acute abdomen. 

## References

[1] Zito PM, Scharf R (2020). Epidermoid Cyst. https://www.ncbi.nlm.nih.gov/books/NBK499974/.

[2] Weir CB, St. Hilaire NJ (2020). Epidermal Inclusion Cyst. https://www.ncbi.nlm.nih.gov/books/NBK532310/.

[3] Park JY, Kim YW, Lee KY, Sung JY (2015). Epidermoid cyst of the cecum. Ann Coloproctol.

[4] Demır H, Aydoğan B, Şahın H, Öcal P, Ilvan Ş (2012). Epidermoid cyst of the cecum: a case report. Turk J Gastroenterol.

[5] Fernández-Castroagudín J, Bustamante Montalvo M, Delgado Blanco M, González-Quintela A, Pintos Martínez E, Varo Pérez E. (2001). Quiste epidermoide: una causa infrecuente de patología quística hepática [Epidermoid cyst: a rare cause of cystic liver disease]. Gastroenterol Hepatol.

[6] Salimi J, Karbakhsh M, Dolatshahi S, Ahmadi SA (2004). Cystic teratoma of the pancreas: a case report. Ann Saudi Med.

[7] Sahoo MR, Gowda MS, Behera SS (2013). Unusual site and uncommon presentation of epidermoid cyst: a rare case report and review of literature. BMJ Case Rep.

[8] Pear BL, Wolff JN (1969). Epidermoid cyst of the cecum. JAMA.

[9] Rahbar M (2018). Appendix epidermoid cyst: presenting as an acute appendicitis. Clin Case Rep.

[10] Cotton MH, Blake JR (1986). Dermoid cyst: a rare tumour of the appendix. Gut.

[11] Piserchia NE, Davey RB (1980). Epidermoid cyst of the appendix. J Pediatr Surg.

[12] Caratozzolo E, Massani M, Recordare A, Ciardo L, Antoniutti M, Jelmoni A, Bassi N (2001). Squamous cell liver cancer arising from an epidermoid cyst. J Hepatobiliary Pancreat Surg.

